# Gestational Diabetes Mellitus and Biomarker Profiles: A BMI-Stratified Analysis of Gremlin 1 and BMP 4—A Cross-Sectional Study

**DOI:** 10.3390/diagnostics16030417

**Published:** 2026-02-01

**Authors:** Yeşim Şalcioğlu, Medeni Arpa, Kübra Sönmez, Şenol Şentürk

**Affiliations:** 1 Department of Medical Biochemistry, Faculty of Medicine, Recep Tayyip Erdoğan University, 53100 Rize, Türkiye; salciogluyesim@gmail.com (Y.Ş.); kubra.sonmez@erdogan.edu.tr (K.S.); 2 Department of Obstetrics and Gynecology, Faculty of Medicine, Recep Tayyip Erdoğan University, 53100 Rize, Türkiye; senol.senturk@erdogan.edu.tr

**Keywords:** gestational diabetes mellitus, Gremlin 1, BMP 4, body mass index, pregnancy, biomarkers

## Abstract

**Background/Objectives**: We sought to examine serum concentrations of Gremlin 1 and BMP 4 in pregnant women diagnosed with gestational diabetes mellitus (GDM) compared to healthy pregnant controls while also exploring potential associations with body mass index (BMI) and gestational age. **Methods**: Our cohort comprised 72 pregnant women—35 with GDM and 37 healthy controls. We measured serum levels of Gremlin 1 and BMP 4 and stratified participants according to BMI categories. Statistical comparisons employed appropriate tests for group differences, and we used Spearman’s correlation to evaluate relationships among BMI, gestational age, fetal birth weight, HOMA-IR, Triglyceride-Glucose Index (TyG), QUICKI and biomarker levels. **Results**: BMI, triglyceride, HOMA-IR, and TyG were significantly higher, and QUICKI was lower in the GDM group compared with controls. Although Gremlin 1 levels were lower and BMP 4 levels and fetal birth weight were higher in the GDM group, these differences were not statistically significant. In BMI stratified analysis, both biomarkers were higher in the normal weight group, without significant differences. BMI correlated negatively with Gremlin 1 and BMP 4, and gestational age correlated negatively with both biomarkers. A strong positive correlation was observed between Gremlin 1 and BMP 4. **Conclusions**: The biomarker patterns observed in GDM appear distinct from those reported in diabetes mellitus, possibly reflecting pregnancy-related physiological weight gain and shifts in body composition. The strong positive relationship between Gremlin 1 and BMP 4 lends support to the notion of coordinated regulatory pathways, potentially indicating cellular resistance to BMP 4’s pro-adipogenic actions. Larger longitudinal investigations incorporating detailed body composition assessments will be essential to elucidate their roles in gestational metabolic adaptations and their potential utility for GDM risk stratification.

## 1. Introduction

Gestational diabetes mellitus (GDM) represents a form of glucose intolerance that emerges or is first identified during pregnancy, resulting in maternal hyperglycemia [1]. This condition typically manifests in the second or third trimester and carries increased risks for adverse maternal and neonatal outcomes, as well as long-term metabolic complications affecting both mother and child [2]. The global prevalence of GDM varies widely, and direct comparisons are difficult due to different screening and diagnostic criteria [3]. The underlying pathophysiology encompasses pregnancy-induced insulin resistance coupled with β-cell dysfunction, culminating in disrupted glucose homeostasis and accompanying inflammatory alterations [4].

Gremlin 1 functions as an antagonist of bone morphogenetic proteins (BMPs) and participates in processes including insulin resistance, adipogenesis, and vascular remodeling [5]. BMP 4, a member of the transforming growth factor β superfamily, regulates adipocyte differentiation and energy metabolism [6]. The BMP family encompasses more than 20 structurally related proteins exhibiting diverse and occasionally opposing metabolic functions. Whereas BMP 4 facilitates white adipocyte differentiation and has been associated with insulin resistance in obesity and type 2 diabetes [7], other family members such as BMP-7 demonstrate protective properties by promoting brown adipose tissue development and enhancing insulin sensitivity [8,9]. This functional heterogeneity within the BMP family highlights the complexity inherent in BMP signaling during metabolic regulation. Altered circulating levels of these proteins have been reported in obesity, type 2 diabetes, and cardiovascular disease [10]; however, their involvement in GDM remains poorly characterized. Elucidating these connections could yield fresh insights into disease mechanisms and uncover potential targets for early detection or therapeutic intervention.

The objective of this research was to evaluate serum Gremlin 1 and BMP 4 concentrations in pregnant women with GDM compared with healthy controls, and to determine their clinical significance in GDM pathogenesis.

## 2. Materials and Methods

### 2.1. Study Population and Ethnicity

This study was conducted in full compliance with the Declaration of Helsinki and received approval from the Institutional Review Board (Ethics Committee) of Recep Tayyip Erdoğan University Faculty of Medicine (Approval number: 2024/200; approval date: 23 July 2024) at the Department of Obstetrics and Gynecology, Recep Tayyip Erdoğan University Training and Research Hospital. Our study population consisted exclusively of women of Turkish ethnicity from the Eastern Black Sea region.

We recruited pregnant women either previously diagnosed with GDM or newly identified during routine antenatal care, along with healthy pregnant women, all of whom provided written informed consent following comprehensive explanation of study objectives, procedures, potential risks, and anticipated benefits. Participants received assurance of their right to withdraw at any time without compromising their clinical care. We maintained strict patient confidentiality throughout, and all data underwent anonymization before analysis.

Our final cohort included 72 women: 35 in the GDM group and 37 in the control group. We classified participants by GDM status and further stratified them according to body mass index (BMI) into normal weight (18–24.9 kg/m^2^) and overweight/obese (≥25 kg/m^2^) categories.

Inclusion criteria encompassed: singleton pregnancy, gestational age between 24 and 28 weeks at diagnosis, age ≥ 18 years, absence of systemic disease, and no prior diabetes diagnosis before pregnancy.

Exclusion criteria comprised: pre-existing type 1 or type 2 diabetes, hypertension, endocrine disorders, alcohol or tobacco use, medications affecting insulin metabolism, multiple pregnancy, and age < 18 years.

### 2.2. Sample Collection and Biochemical Analysis

We retrieved participant data from the laboratory information system. Following routine biochemical testing, residual serum samples were transferred to sterile Eppendorf tubes and stored at −80 °C until analysis. Serum BMP 4 and Gremlin 1 concentrations were quantified using commercial ELISA kits (BT LAB, Shanghai, China; BMP 4: E1990Hu, Gremlin 1: E2735Hu) following the manufacturer’s protocols. Results are expressed as ng/mL for Gremlin 1 and pg/mL for BMP 4. Serum insulin levels were determined by chemiluminescent immunoassay on an ADVIA Centaur XPT analyzer (Siemens Healthineers, Erlangen, Germany). Serum glucose, HDL-C, total cholesterol (TC), and triglyceride (TG) concentrations were measured via colorimetric/photometric methods on an AU680 analyzer (Beckman Coulter, Brea, CA, USA).

### 2.3. Statistical Analysis

Data analysis was conducted using IBM SPSS Statistics software (version 29.0.2.0; IBM Corp., Armonk, NY, USA). Normality was assessed by examining the distribution of each variable. Normally distributed data were evaluated with Independent Sample *t*-test and expressed as mean ± standard deviation (SD), whereas non-normally distributed data were assessed with Mann–Whitney-U test and presented as median (minimum–maximum). Distributions of categorical variables were compared using the chi-square test and reported as percent (%). Relationships between variables were explored using Spearman’s rank correlation. Statistical significance was defined as a *p* value less than 0.05.

## 3. Results

### 3.1. Comparison Between the GDM and Control Groups

Our total sample comprised 72 pregnant women: 35 with GDM (48.6%) and 37 healthy controls (51.4%). Mean ages were comparable between groups, as were gestational ages at sampling. The cesarean section rate reached 57.1% in the GDM group versus 40.5% in healthy controls, a difference that did not attain statistical significance (*p* = 0.159). The cesarean section rate was 57.1% in the GDM group and 40.5% in the healthy control group, and the difference was not statistically significant (*p* = 0.159).

BMI proved significantly higher in the GDM group relative to controls. Serum Gremlin 1 concentrations appeared modestly reduced in women with GDM compared with controls, though this difference lacked statistical significance. Conversely, serum BMP 4 levels showed an increase in the GDM group that similarly failed to reach significance. HOMA-IR and Triglyceride Glucose Index (TyG) values were markedly elevated in the GDM group compared with controls. By contrast, QUICKI—which inversely reflects insulin resistance—was significantly lower in the GDM group. These observations confirm the anticipated metabolic disturbances characteristic of GDM across multiple validated insulin resistance indices. Fetal birth weight was higher in the GDM group (mean difference = 221.4 g) but did not achieve statistical significance. Regarding lipid profiles, TG levels were significantly elevated in the GDM group relative to controls. TC showed a non-significant elevation in the GDM group (mean difference = 9.35 mg/dL). HDL-C and LDL-C levels remained comparable between groups. The data are presented in Table 1.

### 3.2. Comparison According to BMI Categories

When participants were stratified by BMI, 37 women (51.4%) had BMI < 25 kg/m^2^ (normal weight) and 35 women (48.6%) had BMI ≥ 25 kg/m^2^ (overweight/obese). Age and gestational week were similar between BMI categories. Median Gremlin 1 levels tended to be higher in the overweight/obese group compared with the normal weight group, although the difference did not reach statistical significance. Similarly, BMP 4 concentrations were comparable between BMI categories. The data are shown in Table 2.

### 3.3. Correlation Analysis

**Figure 1:**
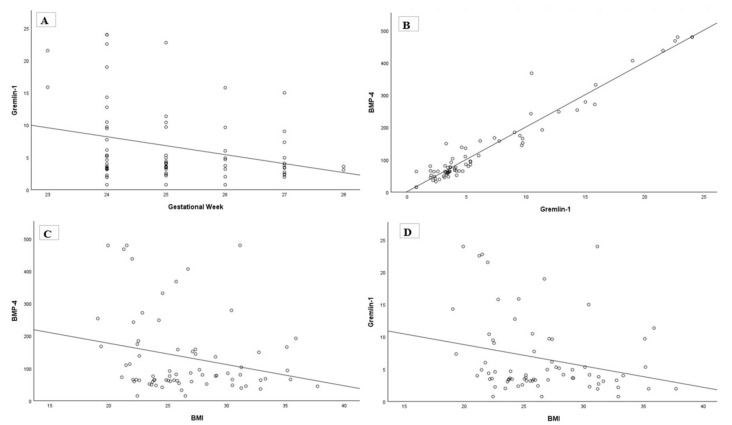
Scatter plots demonstrating correlations between Gremlin 1, BMP 4, gestational week, and BMI. (**A**) Gremlin 1 levels decreased with advancing gestational week. (**B**) Gremlin 1 positively correlated with BMP 4. (**C**) BMP 4 was inversely correlated with BMI. (**D**) Gremlin 1 was inversely correlated with BMI.

As illustrated in Figure 1, Spearman’s correlation analysis revealed a significant negative correlation between BMI and serum Gremlin 1 levels (ρ = −0.244, *p* = 0.039) as well as between BMI and BMP 4 concentrations (ρ = −0.234, *p* = 0.048). Gestational week showed a significant negative correlation with both Gremlin 1 (ρ = −0.290, *p* = 0.014) and BMP 4 (ρ = −0.329, *p* = 0.005). A strong positive correlation was observed between Gremlin 1 and BMP 4 levels (ρ = 0.970, *p* < 0.001).

Neither Gremlin 1 nor BMP 4 showed significant correlations with any of the insulin resistance markers (HOMA-IR, TyG, QUICKI) or fetal birth weight. Specifically, Gremlin 1 showed weak negative correlations with HOMA-IR (ρ = −0.153, *p* = 0.201) and TyG (ρ = −0.030, *p* = 0.803), a weak positive correlation with QUICKI (ρ = 0.129, *p* = 0.281), and a weak negative correlation with fetal birth weight (ρ = −0.120, *p* = 0.315), none of which reached statistical significance. Similarly, BMP 4 demonstrated no significant associations with HOMA-IR (ρ = −0.099, *p* = 0.407), TyG (ρ = −0.025, *p* = 0.833), QUICKI (ρ = 0.085, *p* = 0.475), or fetal birth weight (ρ = −0.118, *p* = 0.323).

When potential associations between Gremlin 1 and BMP 4 with lipid parameters were evaluated, Gremlin 1 showed no significant associations with HDL-C (ρ = −0.011, *p* = 0.929), TC (ρ = −0.099, *p* = 0.407), LDL-C (ρ = −0.043, *p* = 0.719), or TG (ρ = −0.046, *p* = 0.700). Similarly, BMP 4 demonstrated no significant correlations with HDL-C (ρ = −0.024, *p* = 0.842), TC (ρ = −0.092, *p* = 0.442), LDL-C (ρ = −0.017, *p* = 0.890), or TG (ρ = −0.048, *p* = 0.686).

## 4. Discussion

Diabetes mellitus is a chronic metabolic disorder characterized by insufficient insulin production or impaired insulin action, with steadily increasing prevalence over the past decade, making it a major public health concern [11]. GDM, defined as glucose intolerance of varying severity first recognized during pregnancy, typically develops in the second or third trimester as a consequence of pregnancy-induced insulin resistance and β-cell dysfunction. This physiological adaptation aims to ensure adequate glucose delivery to the developing fetus; however, when dysregulated, it produces maternal hyperglycemia [12,13].

Gremlin 1, functioning as a BMP 4 antagonist, exhibits overexpression in obesity and type 2 diabetes and has been implicated in adipose tissue metabolism, insulin resistance, and inflammatory pathways [14]. Although both Gremlin 1 and BMP 4 have been implicated in metabolic disorders, to our knowledge no previous study has simultaneously examined their circulating levels in women with GDM. Our findings therefore provide novel data addressing this gap in the literature.

### 4.1. Metabolic Characterization and Insulin Resistance Markers

The GDM group in our study exhibited significantly higher BMI than the healthy control group. To comprehensively characterize the metabolic phenotype of our cohort, we assessed multiple insulin resistance markers. As anticipated, HOMA-IR was markedly elevated in the GDM group, confirming the central role of insulin resistance in GDM pathophysiology. HOMA-IR has undergone extensive validation as a reliable indicator of insulin resistance during pregnancy and demonstrates strong predictive value for GDM development [15]. Our findings align with previous investigations showing that elevated HOMA-IR in early pregnancy associates with increased GDM risk and adverse pregnancy outcomes [16].

We also evaluated the TyG index, a straightforward surrogate marker of insulin resistance calculated from fasting triglycerides and glucose. The TyG index proved significantly higher in the GDM group, consistent with recent evidence supporting its utility in GDM screening and risk assessment [17]. Studies have demonstrated that early pregnancy TyG index can predict GDM development with comparable or superior performance relative to traditional markers [18]. Additionally, QUICKI—which inversely correlates with insulin resistance—was significantly lower in the GDM group. The concordance across these three distinct insulin resistance indices strengthens confidence in our metabolic characterization and confirms the expected insulin resistance phenotype in GDM.

### 4.2. Lipid Metabolism and Dyslipidemia

Analysis of lipid parameters revealed significant hypertriglyceridemia in the GDM group, while total cholesterol, HDL-C, and LDL-C did not differ significantly between groups. Hypertriglyceridemia is a well-established feature of GDM, reflecting the combined effects of insulin resistance, increased hepatic VLDL production, and decreased lipoprotein lipase activity [19]. Elevated maternal triglyceride levels have been linked to pregnancy complications, including preeclampsia and excessive fetal growth. The physiological hyperlipidemia of pregnancy, which serves to support fetal growth and development, becomes exaggerated in GDM due to enhanced insulin resistance and altered lipid metabolism [20].

Interestingly, despite the significant differences in triglycerides and the established relationship between Gremlin 1 and lipid metabolism reported by Deischinger et al., neither Gremlin 1 nor BMP 4 showed significant correlations with any lipid parameters in our cohort. This discrepancy could be attributed to a variety of elements. First, the relationship between Gremlin 1 and lipid metabolism may be more prominent in the postpartum period or in the context of hepatic steatosis rather than during active pregnancy [14]. Second, the physiological hyperlipidemia of pregnancy may obscure subtle biomarker-lipid relationships that might be apparent in non-pregnant states. Third, Gremlin 1’s effects on lipid metabolism may be mediated through tissue-specific mechanisms not reflected in circulating lipid concentrations.

### 4.3. Gremlin 1 and BMP 4

Although serum Gremlin 1 levels appeared slightly lower and BMP 4 levels appeared higher in the GDM group, these differences failed to achieve statistical significance. Baboota et al. reported a positive correlation between Gremlin 1 levels and insulin resistance in their investigation of the regulatory effects of BMP 4 and Gremlin 1 on hepatic cell senescence [6]. Similarly, Al Regaiey et al. reported that Gremlin 1 levels were elevated in individuals with type 2 diabetes relative to healthy controls, with the greatest elevations observed in those with poor glycemic control. Gremlin 1 correlated positively with fat mass, visceral adiposity, insulin resistance, HbA1c, and HOMA-IR, supporting its association with adverse metabolic profiles [21].

In contrast, our findings did not demonstrate significant differences in Gremlin 1 or BMP 4 between GDM and control groups, nor did we observe correlations between these biomarkers and established insulin resistance markers. This suggests that the associations observed in type 2 diabetes may not be directly applicable to the gestational context, possibly due to pregnancy-specific metabolic adaptations. The absence of correlations between Gremlin 1/BMP 4 and multiple validated insulin resistance indices strengthens the conclusion that these biomarkers may not directly reflect metabolic dysfunction in late pregnancy GDM.

Deischinger et al. demonstrated that Gremlin 1 levels during pregnancy and the postpartum period were related to hepatic steatosis index, bone health parameters, glucose metabolism, and pregnancy status [14]. However, they found no significant difference in Gremlin 1 concentrations between women with GDM and those with normal glucose tolerance. In their measurements at 24–28 weeks of gestation, Gremlin 1 levels were higher in the normal glucose tolerance group, but the difference was not statistically significant. The absence of significant differences in Gremlin 1 and BMP 4 levels between our GDM and control groups is consistent with these findings. This concordance may be explained by factors such as sample size, timing of biomarker measurement, and biological variability. Moreover, although most previous studies have reported a positive association between Gremlin 1, insulin resistance, and adiposity, the physiological changes in pregnancy may attenuate or mask these relationships.

Our findings warrant interpretation within the context of our study population’s ethnic composition. All participants were of Turkish ethnicity from the Eastern Black Sea region, representing a relatively homogeneous population. Biomarker profiles, including Gremlin 1 and BMP 4, may exhibit different patterns across ethnic groups owing to genetic variations, dietary habits, lifestyle factors, and baseline metabolic characteristics. For instance, insulin resistance markers and lipid profiles show well-documented ethnic variations, with different thresholds for metabolic risk across populations [22]. Asian populations demonstrate higher insulin resistance and GDM risk at lower BMI thresholds compared with Caucasian populations, while African and Hispanic populations exhibit distinct metabolic profiles. Whether the lack of association between Gremlin 1/BMP 4 and metabolic parameters in our Turkish cohort reflects population-specific patterns or represents a universal finding in GDM requires validation in diverse ethnic groups. Notably, our findings align with those of Deischinger et al. [14], who studied a predominantly Caucasian Austrian population and similarly found no significant differences in Gremlin 1 between GDM and control groups. This concordance across two different European populations (Turkish and Austrian) suggests that the absence of Gremlin 1 differences in GDM may hold consistent across Caucasian populations.

### 4.4. Pregnancy Outcomes

To evaluate the clinical relevance of our findings, we analyzed pregnancy outcomes including delivery mode and fetal birth weight. Cesarean section rates were numerically higher in the GDM group, though this did not reach statistical significance. This trend is consistent with the known increased risk of operative delivery in GDM pregnancies due to factors such as macrosomia, failed induction, and clinical management protocols.

Fetal birth weight showed a borderline significant increase in the GDM group, suggesting a trend toward fetal overgrowth. This finding aligns with the well-established association between maternal hyperglycemia and excessive fetal growth mediated through increased transplacental glucose transfer and fetal hyperinsulinemia [23,24]. Maternal hyperglycemia leads to fetal hyperinsulinemia, which acts as a growth factor promoting increased fat deposition and macrosomia [23]. The borderline significance in our study may reflect adequate glycemic control in our GDM cohort or limited sample size to detect this effect definitively.

Importantly, neither Gremlin 1 nor BMP 4 showed associations with delivery mode or fetal birth weight. This lack of correlation with clinical outcomes, combined with the absence of associations with metabolic and lipid parameters, suggests that these biomarkers may not serve as predictors of pregnancy complications in late-stage GDM. Several factors could account for the divergence observed between biomarker levels and clinical outcomes: these biomarkers may play roles in early GDM pathogenesis rather than late pregnancy complications, their effects may be mediated through pathways not captured by standard clinical assessments, or the timing of measurement may not coincide with their peak clinical relevance.

### 4.5. BMI, Body Composition

Both Gremlin 1 and BMP 4 concentrations appeared higher in the normal-weight group, although the differences lacked statistical significance. Gremlin 1 is known to be highly expressed in human adipose tissue, with levels particularly elevated in hypertrophic obesity [25]. As a secreted antagonist protein, it inhibits BMP 4 activity on preadipocytes, thereby modulating adipogenesis. Increased Gremlin 1 expression has been reported in obese individuals, with the highest levels in patients with type 2 diabetes [10]. Hedjazifar et al. further demonstrated that Gremlin 1 antagonizes insulin signaling, reduces glucose-mediated responses, and is more abundant in visceral than subcutaneous adipose tissue, especially in hypertrophic fat depots [5]. These findings support a positive correlation between Gremlin 1, adiposity, and insulin resistance. In our study, however, no significant differences in Gremlin 1 or BMP 4 were observed between BMI categories, suggesting that pregnancy-related metabolic changes may influence these associations.

The accuracy and clinical utility of body composition assessment methods differ considerably, which carries important implications for interpreting adiposity-related biomarkers [26]. In our cohort, mean BMI in both groups exceeded 25 kg/m^2^, placing most participants in the overweight category, which may have contributed to the absence of significant differences in Gremlin 1 levels between groups. Physiological weight gain and altered body composition during pregnancy could produce different biological patterns compared with non-pregnant populations, potentially explaining the slightly higher Gremlin 1 levels observed in healthy pregnant women. Body composition was not directly assessed using methods such as bioelectrical impedance analysis, which may have limited interpretation of adiposity-related biomarker changes.

Correlation analysis revealed weak but statistically significant negative associations between BMI and both Gremlin 1 and BMP 4, indicating a tendency for these biomarker levels to decrease as BMI increases. This result is consistent with the negative association between BMI and Gremlin 1 reported by Deischinger et al. Taken together, our findings suggest that elevated BMI may attenuate circulating Gremlin 1 and BMP 4 levels, with pregnancy-specific adaptations further shaping these relationships.

BMP 4 serves as a key regulator of adipogenesis, particularly influencing white adipose tissue. In a study of hypertrophic obesity, the expression of BMP 4 and its antagonists was evaluated, demonstrating that BMP 4 secretion was markedly increased in obesity [27]. Similarly, Baboota et al. [6] and Wang et al. [28] reported significant positive correlations between BMP 4 levels and total body fat percentage, highlighting its association with adiposity. However, these studies were conducted in non-pregnant populations. Considering that physiological weight gain during pregnancy alters body composition in distinct ways, the higher BMP 4 levels observed in pregnant women with BMI < 25 kg/m^2^ in our study may reflect such pregnancy-specific adaptations. This suggests that metabolic regulatory mechanisms active during gestation could modulate the relationship between BMI and BMP 4, potentially explaining the absence of significant differences in our cohort.

### 4.6. Gestational Age

In this study, advancing gestational age was associated with a statistically significant decline in serum Gremlin 1 and BMP 4 concentrations. This finding aligns with the results of Deischinger et al., who reported lower Gremlin 1 levels as pregnancy progressed, with values before 20 weeks exceeding those measured at 24–28 weeks of gestation. We also observed a strong positive correlation between Gremlin 1 and BMP 4, in agreement with Baboota et al. [6], who demonstrated concurrent increases in the expression of both molecules in non-pregnant populations with fatty liver disease and type 2 diabetes. Experimental models have shown that Gremlin 1 can inhibit BMP 4, highlighting the dynamic interplay between these two factors [29,30]. Increased gene expression of BMP 4 and its antagonists in hypertrophic obesity has been reported, with Gremlin 1 levels positively correlated with BMP 4 and adipocyte size [25,31]. Taken together, the concurrent elevation of BMP 4 and its inhibitor Gremlin 1 may reflect a potential cellular resistance mechanism to the pro-adipogenic effects of BMP 4. Our findings align with this framework, suggesting that similar regulatory dynamics may occur during pregnancy, potentially influenced by gestational metabolic adaptations.

### 4.7. Limitations

This study has several limitations that should be considered when interpreting the findings. First, the sample size was relatively small, which may have limited the statistical power to detect subtle differences in biomarker levels between groups. However, our ability to detect robust differences in established metabolic markers (HOMA-IR, TyG, QUICKI, triglycerides) suggests adequate power for identifying clinically meaningful changes, strengthening confidence that the absence of Gremlin 1 and BMP 4 differences reflects true biological patterns. Additionally, our study population consisted exclusively of Turkish women from a single geographic region, which limits generalizability to other ethnic and racial groups. Given that biomarker profiles and metabolic parameters can vary significantly across populations, our findings require validation in diverse ethnic cohorts. Second, the cross-sectional design precludes assessment of causal relationships between Gremlin 1, BMP 4, and metabolic parameters. Third, body composition was not directly measured using techniques such as bioelectrical impedance analysis or dual-energy X-ray absorptiometry, which could have provided more precise information on adiposity and fat distribution. Fourth, biomarker measurements were performed at a single gestational time point, and longitudinal changes throughout pregnancy were not evaluated. Finally, potential confounding factors such as dietary intake, physical activity, and inflammatory status were not assessed, which may have influenced circulating Gremlin 1 and BMP 4 concentrations. Future studies with larger, longitudinal cohorts and comprehensive metabolic profiling are warranted to validate and expand upon these findings.

## 5. Conclusions

This study provides a comprehensive evaluation of serum Gremlin 1 and BMP 4 levels in women with GDM, incorporating detailed metabolic, lipid, and clinical outcome assessments. Our robust metabolic characterization successfully identified expected GDM-related changes: significantly elevated insulin resistance markers (HOMA-IR, TyG index, reduced QUICKI) and hypertriglyceridemia, alongside trends toward increased fetal birth weight. These findings validate our methodological approach and confirm our ability to detect clinically meaningful metabolic differences.

Gremlin 1 and BMP 4 levels showed no significant differences between GDM and control groups and demonstrated no correlations with insulin resistance markers, lipid parameters, or pregnancy outcomes. The consistency of these null findings across multiple metabolic domains is informative rather than limiting. It indicates that these biomarkers operate through pathways distinct from the insulin resistance and lipid dysregulation that characterize late pregnancy GDM. The strong positive correlation between Gremlin 1 and BMP 4, combined with their negative associations with BMI and declining levels with gestational age, reveals coordinated regulation that warrants mechanistic investigation.

An important consideration in interpreting our BMI-related findings is that pregnancy-specific weight gain reflects not only maternal adiposity but also fetal weight, placental mass, amniotic fluid, and expanded blood volume. This physiological complexity may obscure relationships between BMI and adiposity-related biomarkers that are apparent in non-pregnant populations. The absence of direct body composition assessment in our study limits precise evaluation of maternal fat mass versus pregnancy-related weight components. Future studies incorporating bioelectrical impedance analysis or other body composition methods would better clarify whether Gremlin 1 and BMP 4 relate specifically to maternal adiposity independent of pregnancy-related weight gain.

Our findings contribute important insights to GDM biomarker research. The biomarker profile in GDM appears fundamentally different from type 2 diabetes, reflecting pregnancy-specific metabolic adaptations and the unique challenge of distinguishing maternal adiposity from physiological gestational weight gain. Rather than serving as markers of established metabolic dysfunction, Gremlin 1 and BMP 4 may play roles in early pathogenic processes, tissue-specific regulation, or adaptive responses to pregnancy that are not captured by third-trimester serum measurements or conventional anthropometric indices. Our comprehensive metabolic profiling establishes a foundation for future mechanistic studies exploring when and how these biomarkers function in gestational metabolism.

These results guide future research directions. Longitudinal studies measuring biomarkers from early pregnancy through postpartum, combined with detailed body composition analysis, will clarify their temporal dynamics and relationships to true maternal adiposity versus pregnancy-related weight changes. Mechanistic investigations examining tissue-specific expression, inflammatory pathways, and adipokine networks will elucidate the biological roles of Gremlin 1 and BMP 4 in pregnancy. Our work demonstrates that understanding biomarker function in pregnancy requires moving beyond simple case–control comparisons and conventional BMI assessments to embrace the complexity of gestational physiology and body composition changes.

## Figures and Tables

**Table 1:** Comparative Analysis of Demographic, Fetomaternal, and Biomarker Profiles Between Groups.

	Healthy Control (*n* = 37)	GDM (*n* = 35)	*p* Value ^a^
Age (years)	30 ± 5	31 ± 6	0.324
BMI (kg/m^2^)	25.32 ± 3.20	27.70 ± 5.12	0.022
Gestational Week	25 ± 1	25 ± 1	0.094
Gremlin 1 (ng/mL)	4.12 (2–24)	3.81 (0.75–24)	0.191
BMP 4 (pg/mL)	77.5 (32.35–480)	80.2 (15–480)	0.464
TG (mg/dL)	87.16 ± 39.30	121.77 ± 83.80	0.015
TC (mg/dL)	163.14 ± 34.78	172.49 ± 35.10	0.130
HDL-C (mg/dL)	57.27 ± 11.79	52.74 ± 12.83	0.123
LDL-C (mg/dL)	88.38 ± 24.85	95.31 ± 30.09	0.144
Fetal Birth Weight (g)	3235 ± 426	3456 ± 529	0.054
HOMA-IR	1.54 ± 0.57	2.72 ± 0.96	<0.001
TyG	8.03 ± 0.46	8.40 ± 0.66	0.004
QUICKI	0.492 ± 0.045	0.428 ± 0.041	<0.001

BMP 4: Bone Morphogenetic Protein-4; BMI: Body Mass Index; TG: Triglyseride; TC: Total Cholesterol; HDL-C: High Density Lipoprotein Cholesterol; LDL-C: Low Density Lipoprotein Cholesterol; HOMA-IR: InsulinxGlucose/405; TyG: ln^TGxGlucose/2^; QUICKI: 1/(log^Insulin^ + log^Glucose^). Data were reported as mean ± standard deviation or median (minimum–maximum) according to their distribution. ^a^: Independent Samples *t*-test or Mann–Whitney U test.

**Table 2:** Demographic Profiles and Circulating Biomarkers in the BMI groups.

	Normal Weight (*n* = 37)	Overweight/Obese (*n* = 35)	*p* Value ^a^
Age (years)	29 ± 5	32 ± 6	0.079
Gestational Week	25 ± 1	25 ± 1	0.359
Gremlin 1 (ng/mL)	4.62 (0.75–24)	3.75 (0.75–24)	0.147
BMP 4 (pg/mL)	92.7 (15–480)	78.7(15–480)	0.275

BMP 4: Bone Morphogenetic Protein-4; BMI: Body Mass Index; Data were reported as mean ± standard deviation or median (minimum–maximum) according to their distribution. ^a^: Independent Samples *t*-test or Mann–Whitney U test.

## Data Availability

The original contributions presented in this study are included in the article. Further inquiries can be directed to the corresponding author.
